# In‐Depth Comparative Study of the Cathode Interfacial Layer for a Stable Inverted Perovskite Solar Cell

**DOI:** 10.1002/cssc.202100585

**Published:** 2021-05-07

**Authors:** Jinho Lee, Harun Tüysüz

**Affiliations:** ^1^ Max-Planck-Institut für Kohlenforschung Kaiser-Wilhelm-Platz 1 45470 Mülheim an der Ruhr Germany; ^2^ Department of Physics Incheon National University 119 Academy-ro, Yeonsu-gu Incheon 22012 Republic of Korea

**Keywords:** electron transport layer, metal oxides, perovskites, solar cells, titanium

## Abstract

Achieving long‐term device stability is one of the most challenging issues that impede the commercialization of perovskite solar cells (PSCs). Recent studies have emphasized the significant role of the cathode interfacial layer (CIL) in determining the stability of inverted p‐i‐n PSCs. However, experimental investigations focusing on the influence of the CIL on PSC degradation have not been systematically carried out to date. In this study, a comparative analysis was performed on the PSC device stability by using four different CILs including practical oxides like ZnO and TiO_*x*_. A new implemented co‐doping approach was found to results in high device performance and enhanced device stability. The PSC with a thick film configuration of chemically modified TiO_*x*_ CIL preserves over 77 % of its initial efficiencies of 17.24 % for 300 h under operational conditions without any encapsulation. The PSCs developed are among the most stable reported for methylammonium lead iodide (MAPbI_3_) perovskite compositions.

## Graphical abstract

**In for the CIL**: Highly stable inverted perovskite solar cells (PSCs) are assembled by using chemically modified titanium suboxide (TiO_*x*_) as the cathode interfacial layer (CIL). Codoping enhances the electrical properties of the TiO_*x*_ CIL, providing better inner encapsulation with thick film configuration. The PSCs exhibit significantly improved operational stability, maintaining >77 % of their initial efficiencies for up to 300 h without encapsulation.

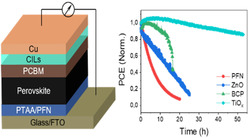

## Introduction

Perovskite solar cells (PSCs) have emerged as promising next‐generation energy technology, owing to advantageous features such as solution processability and high power per weight value as well as enabling fabrication of ultralight solar module via high throughput printing process.[^1^, ^2^] The unique optoelectronic properties, such as low exciton binding energy,[^3^, ^4^] long exciton lifetime[^5^, ^6^, ^7^] and diffusion length (>1 μm),[^8^, ^9^, ^10^] high extinction coefficient (ca. 10^5^ cm^−1^),[^4^, ^11^] tunable bandgap,[^12^, ^13^, ^14^, ^15^, ^16^, ^17^] and high ambipolar charge carrier mobility,[^18^, ^19^] along with intensive research efforts enable significant progress in their power conversion efficiencies (PCEs), reaching a certified PCE up to 25.5 %.^[20]^ Despite this encouraging progress, high‐efficiency PSCs continue to present several challenges including operational stability and compatibility with a high‐throughput continuous printing process for future large‐area application.[^21^, ^22^, ^23^, ^24^, ^25^, ^26^, ^27^] Although conventional PSCs with a regular n‐i‐p structure that typically consisted of titanium dioxide (TiO_2_) have so far provided record efficiencies, high processing temperature (ca. 450 °C) and photocatalytic activity of TiO_2_ are the potential drawbacks for the commercial applications.[^28^, ^29^, ^30^, ^31^, ^32^] The recent development of tin oxide (SnO_2_) has significantly lowered the process temperature of regular type PSCs, but it is still close to 200 °C, which limits the flexibility to PSCs.[^33^, ^34^, ^35^]

In this respect, many studies have been conducted on the development of device configurations including inverted p‐i‐n structure comprised of stacked planar thin films since ambipolar carrier transport properties with a long diffusion length of perovskite materials ensures efficient charge collection even in the thick film.[^36^, ^37^, ^38^, ^39^] An appropriate interface engineering should be introduced to optimize interfacial characteristics, such as charge transport and surface passivation, both of which are important for constructing efficient and stable PSCs. In planar device architecture, perovskite layers are placed between charge extraction layers that extract either electron or hole carriers to avoid charge recombination without accumulation at the interface. Interfacial layer, particularly a cathode interfacial layer (CIL), in which fullerene derivatives such as [6,6]‐phenyl‐C_61_‐butyric acid methyl ester (PCBM) or C_60_ are used as the electron extraction layer, can be additionally introduced before depositing top electrodes, which provides the improved electrical contact between fullerene and electrode.[^40^, ^41^, ^42^]

More importantly, CIL is able to perform as an inner encapsulation layer which is beneficial for enhancing the stability of PSC device in the following aspects: perovskites are highly susceptible to moisture‐induced decomposition.[^43^, ^44^, ^45^, ^46^] On the other hand, halide ionic defects, known to be inevitable in the perovskite film, are mobile and thus migrate across the PCBM layer, which eventually reacts with conventional metal, resulting in corrosion of interfacial contact.[^47^, ^48^, ^49^] Such degradation pathways could be mitigated by introducing a permeation barrier layer to restrict the diffusion of molecules/ions (e. g., water, oxygen, and halides) while facilitating electron transport without concomitant loss.[^50^, ^51^, ^52^, ^53^, ^54^] Recently, physically robust CILs have been developed based on organic/inorganic multilayers,[^51^, ^52^, ^53^, ^54^] atomic layer deposition (ALD)‐deposited metal oxides,[^55^, ^56^, ^57^] and chemically inert metals, which significantly enhances the stability of the PSCs. However, these technologies have disadvantages in terms of complicated processing steps and highly sophisticated vacuum process, limiting the application of CILs in a continuous printing process. Hence, the development of solution‐processable CIL that concurrently enhances the device stability against external sources as a core function has been a constant challenge. To date, several printable CILs have been incorporated into inverted p‐i‐n PSCs, which have demonstrated high device performance, whereas comparative studies of their device stability have not yet been explored.

Herein, we have conducted a comprehensive investigation on the stability of inverted p‐i‐n PSCs using organic‐ and inorganic‐based CILs, such as poly[(9,9‐bis(3′‐(*N*,*N*‐dimethylamino)propyl)‐2,7‐fluorene)‐*alt*‐2,7‐(9,9‐dioctylfluorene)] (PFN), zinc oxide nanoparticle (ZnO), bathocuproine (BCP), and titanium suboxide (TiO_*x*_). All PSCs exhibited similarly initial high PCEs of around 18 %, but their stability behavior was very different; intrinsic material properties of CILs play an important role in the overall stability. By systematically exploring the degradation process based on the comparative analysis, we found that stable devices were observed to be resistant to the evolution of both interfacial resistance and the trap states. Notably, chemically modified TiO_*x*_ with improved electron‐transporting property effectively isolates the moisture‐sensitive perovskite, which largely suppresses the energetic disorder (e. g., trap state density and distribution) induced by degradation. The stability of the devices can be further enhanced by incorporating polymer‐modified PCBM and a thicker TiO_*x*_ layer, maintaining >77 % of its initial efficiency for 300 h under continuous illumination without encapsulation.

## Results and Discussion

**Figure 1:**
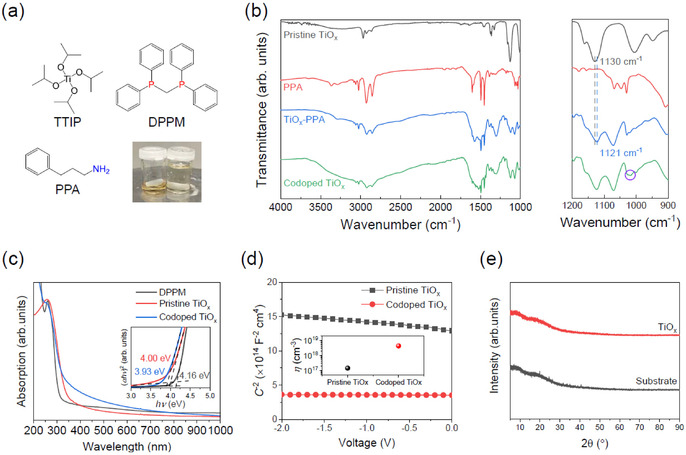
a) Chemical structures of precursor TTIP and reagents DPPM and PPA for chemical doping. The inset displays a photograph of the nitrogen‐mediated stock solution (left) and codoped diluted precursor solution (right). b) FTIR spectra and magnified scanning regions (right) for pristine TiO_x_, PPA, TiO_x_−PPA, and codoped TiO_x_. The marked violet circle in magnified spectrum represents the evolution of a new peak with the addition of DPPM to the TiO_x_−PPA matrix. c) UV/Vis absorption spectra of DPPM, pristine TiO_x_, and codoped TiO_x_. d) Mott‐Schottky plots of the capacitance‐voltage (*C*−*V*) characteristics. e) XRD pattern of the TiO_x_ thin film.

To prepare the set of CIL, we first synthesized precursor solution of TiO_*x*_ and ZnO nanoparticles, as the PFN and BCP are commercially available. Sol‐gel‐based amorphous TiO_*x*_ has been widely used in organic solar cells and PSCs as a CIL because of its low‐temperature solution processability and excellent charge selective behavior.[^49^, ^58^, ^59^, ^60^, ^61^] Figure 1a shows the chemical structures of the materials, titanium(IV) isopropoxide (TTIP), bis(diphenylphosphino)methane (DPPM), and 3‐phenyl‐1‐propylamine (PPA) used for precursor synthesis. The TTIP was initially mixed with PPA to form Ti−N bonds in which lone pair electrons on amine moieties of PPA chemically interact with Ti cations, resulting in N‐doping of the TiO_*x*_. After the doping process, the transparent precursor solution transformed into a low‐density gel with a reddish yellow color. Fourier transform infrared (FTIR) transmission spectra of pristine TiO_*x*_, PPA, and TiO_*x*_−PPA were collected to verify the interactions between the molecules. As seen Figure 1b, the pristine TiO_*x*_ exhibit a characteristic peak at 1129 cm^−1^ assigned to the symmetry stretching vibration of Ti−O−C bond, which is shifted to the lower wavenumber of 1122 cm^−1^ when forming N‐substituted TiO_*x*_ film. This result indicates that Ti−O−C bond weakens, which can be attributed to the formation of N‐related bonds such as Ti−N and N−O. However, as confirmed by the UV/Vis absorption spectra, only very weak additional absorption in the sub‐bandgap region was observable, indicating the minute level of doping (see the Supporting Information, Figure S1). TiO_*x*_ was further doped with phosphorus by adding DPPM into the N‐doped TiO_*x*_ sol‐gel product, in which codoped TiO_*x*_ film is synthesized during the hydrolysis and condensation of N‐doped TiO_*x*_‐containing DPPM. As one of the elements in Group V, phosphorous possesses the same number of valance electrons as nitrogen but has a stronger electron‐donating capability. The resultant redshift of the absorption spectra of the codoped TiO_*x*_ film corresponding to the bandgap narrowing from 4.00 to 3.93 eV is a phenomenon found in P‐doping of titanium dioxides, which is the opposite of the trend in N‐doping (Figure 1c). Although emerging peak at 1018 cm^−1^ that might be associated with the interaction between DPPM and TiO_*x*_ is relatively weak, the presence and interaction (789.5 cm^−1^) of DPPM in the TiO_*x*_‐DPPM film is at a level that can be seen in the FTIR spectra (Figure 1b and Figures S2 and S3). Note that the amount of DPPM in the TiO_*x*_‐DPPM is identical to that of the codoped TiO_*x*_.

To confirm the effect of n‐doping on the carrier density of TiO_*x*_, we measured *C*−*V* characteristics of pristine and codoped TiO_*x*_ films by placing the samples between metal contacts. Figure 1d shows Mott‐Schottky plots (*C* ^−2^ vs. *V*) of the films. The charge carrier density values are calculated according to Equation (1), as obtained from Schottky‐junction theory:

(1) $$\frac{A^{2}}{C^{2}}=\frac{2}{q\epsilon_{0}\epsilon_{r}N}\left(V_{bi}-V\right)$$

where *A* is the active area of the device, *q* is the elementary charge, *ϵ*_0_ is the permittivity in a vacuum, *ϵ*_r_(=9) is the relative permittivity, and *V*_bi_ and *V* refer to built‐in potential and applied voltage, respectively.[^49^, ^62^] The codoped TiO_*x*_ film was modified to have a charge carrier density of 3.6×10^18^ cm^−3^, which is an order of magnitude higher than that of the pristine TiO_*x*_ (1.8×10^17^ cm^−3^), indicating the enhanced n‐type property in codoped TiO_*x*_. To verify the effect of doping on the electrical properties, the *J*−*V* characteristics with a metal/TiO_*x*_/metal device configuration were measured (Figure S4). The codoping of TiO_*x*_ facilitated electron transport with reduced electrical resistance, increasing the current density. In general, TiO_2_ is subjected to high‐temperature calcination to increase its crystallinity for ensuring high electrical conductivity. However, the TiO_*x*_ layer used in this study was annealed at only 100 °C which is far below typical crystallization temperatures of the anatase and rutile phases, exhibiting amorphous feature as confirmed by X‐ray diffraction (XRD) data (Figure 1e). In addition to the sol‐gel processed TiO_*x*_ layer, a dispersed ZnO nanoparticle solution was prepared by using ethanolamine as a dispersing agent, and detailed synthetic procedures are described in the Experimental Section. The size of ZnO nanoparticles was characterized by high‐resolution transmission electron microscopy (HR‐TEM) analysis, revealing the particle size of approximately 15 nm, in which their hydrodynamic diameter distribution is stable against ageing (Figure S5). In addition, ZnO nanoparticle film displayed pronounced XRD characteristics reflections, which match well with the previous results.^[63]^

**Figure 2:**
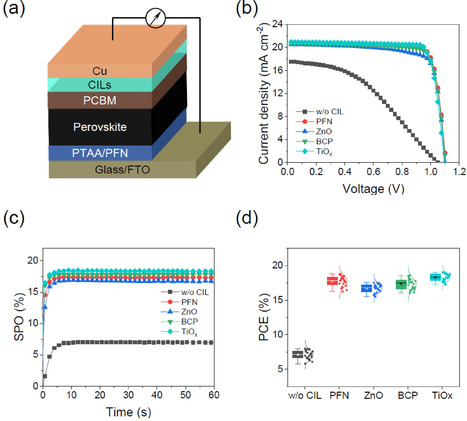
a) Device structure of inverted p‐i‐n PSC. b) *J*−*V* characteristics and c) stabilized power output (SPO), measured for 60 s at a fixed voltage near the maximum power point (MPP) identified in the *J*−*V* characteristics, of PSCs with different CILs. d) Distribution of efficiency for 20 PSC devices fabricated under the same conditions.

To access whether the CILs can improve the device performance, we fabricated the PSCs with p‐i‐n planar heterojunction structure of FTO/poly(triarylamine) (PTAA)/PFN/perovskite/PCBM/CIL/Cu, where the methylammonium lead iodide, MAPbI_3_ was used as a perovskite‐based photoactive layer (Figure 2a and Figure S6). Note that PFN was introduced onto the PTAA to improve the processability of the perovskite layer. Figure 2b shows *J*−*V* characteristics of the PSCs with various CILs and corresponding photovoltaic parameters are summarized in Table 1. Because both PTAA and PCBM perform as excellent charge‐selective contacts, it is highly expected that device performance can be largely affected by the CILs. Considering the energy levels, the working principle of PFN and BCP follows the tunneling mechanism, whereas ZnO and TiO_*x*_ provide favorable contacts for electron transport (Figures S7 and S8).

**Table 1:** Photovoltaic performance parameters of perovskite solar cells with different CILs.

CIL	*V*_OC_ [V]	*J*_SC_ [mA cm^−2^]	FF	PCE [%]
PCBM‐only	1.051	17.58	0.408	7.54
PFN	1.105	20.87	0.814	18.77
ZnO	1.101	20.51	0.782	17.65
BCP	1.104	20.69	0.809	18.49
TiO_*x*_	1.095	20.95	0.832	19.09

Overall, the PSCs with CILs outperform the PCBM‐only PSC that yields an inferior PCE value of 7.54 %, which is attributed to the energy barrier between PCBM and Cu electrode. The PSCs with TiO_*x*_ presented the highest PCE of 19.09 %, with an open‐circuit voltage (*V*_OC_) of 1.095 V, a short‐circuit current density (*J*_SC_) of 20.95 mA cm^−2^ and a fill factor (FF) of 0.832. Compared with TiO_*x*_, the devices with PFN, ZnO, and BCP exhibited slightly lower but almost comparable performance parameters (*V*_OC_, *J*_SC_ and FF), producing PCE values of 18.77 %, 17.65 %, and 18.49 %, respectively. The stabilized power output (SPO) was determined by holding the cell at a fixed voltage near the maximum power point (MPP) voltage over the 60s, as shown in Figure 2c. Regardless of the type of CIL, PSCs rapidly stabilize within 5 s to reach the similar PCE values obtained from current density‐voltage (*J*−*V*) characteristics, reflecting reliable device operation without significant hysteresis. This is because the electron extraction is dominated by the PCBM layer, which appears to be the extent of the quenching of photoluminescence (Figure S9). In Figure 2d, the statistical distribution of the device performances among 20 separated PSCs is presented.

**Figure 3:**
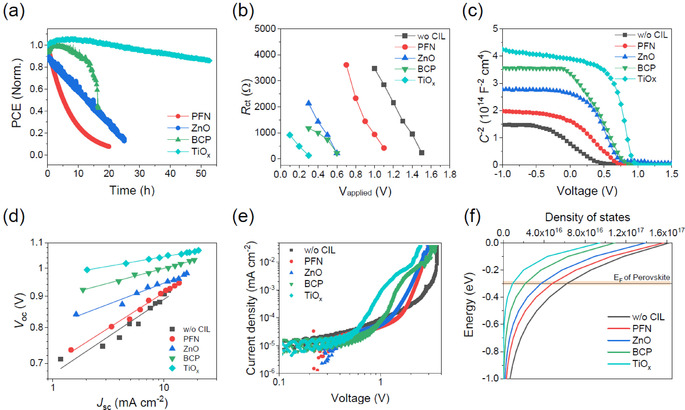
a) Evolution of PCE profiles of non‐encapsulated devices with different CILs under continuous standard illumination in an ambient atmosphere. b) Charge transfer resistance (*R*_ct_) values calculated from impedance spectra for degraded devices. c) Mott‐Schottky plots and d) *V*_OC_ vs. *J*_SC_ for degraded PSCs. e) Dark *J*−*V* characteristics of the electron‐only devices degraded under the same conditions as the device used in Figure b–d. f) Degree of energetic disorder expressed as an intra‐bandgap trap states density and distribution in the PSCs.

We finally investigated the stability of PSCs with different CILs to validate their capability to implement stable devices, as an important subject for commercialization. Figure 3a shows the degradation profiles of the unencapsulated PSCs under operational condition, in which the devices are exposed to continuous light in an ambient atmosphere. The devices with PFN and ZnO CILs rapidly degraded, losing more than half of their initial PCEs after devices were aged over 6 h and 13 h, respectively. The origin of inferior device stability could be different between PFN and ZnO. Owing to the presence of hydrophilic ionic functional groups in its molecular structure, the PFN possesses a weak but distinct hygroscopic nature, which makes it vulnerable to moisture. On the other hand, ZnO in the form of nanoparticles inevitably forms voids between the particles, which can act as a penetration pathway for external molecules such as water and oxygen. From this point of view, similarly rapid reduction in efficiency is observable in the devices without CIL (Figure S10). The BCP significantly retards the efficiency loss of the devices, but still exhibit a sudden decrease in PCE after 15 h of device operation. This result can be attributed to the performance of the BCP CIL as a penetration barrier. Notably, PSC with TiO_*x*_ showed the most stable behavior, preserving roughly 90 % of its initial PCE for up to 40 h. This stability enhancement effect is still valid in PSC with pristine TiO_*x*_ CIL despite its relatively low initial efficiency (Figure S11). We infer that sol‐gel‐based continuous metal‐oxygen networks lead to the formation of the dense film, demonstrating better protective function than other CILs. The trend observed in the air‐stability test is consistent with the operational stability, reflecting the importance of CIL as an inner encapsulation layer (Figure S12).

To gain a deeper understanding of the changes inside the device during the degradation, we conducted an in‐depth comparative study by using degraded samples with different CILs. The analysis was performed by collecting devices that had deteriorated for 10 to 15 h. First, the electrical properties under different applied bias were investigated by extracting internal charge transport resistance (*R*_ct_) from electrochemical impedance spectroscopy measurements. Figure 3b shows the *R*_ct_ values of the degraded PSCs with CILs as a function of applied voltage under dark condition, where the *R*_ct_ is derived from fitting the Nyquist plots of the impedance spectra to an equivalent circuit model (Figure S13). Severely degraded devices (PCBM‐only and PFN) show higher *R*_ct_ compared with those of the stable devices that maintain low *R*_ct_ even under a low applied voltage. We further analyzed the Mott‐Schottky plots of the degraded devices, as shown in Figure 3c. Generally, the built‐in potential (*V*_bi_) can be estimated from the intercept of the linear regime with the x‐axis in the Mott‐Schottky plots. The decrease in *V*_bi_ for degraded PSCs implies the substantial alteration of energetic properties at the interface between the perovskite and CILs as a consequence of interfacial charge accumulation. In addition, the drop in *V*_bi_ associated with *V*_OC_, which is not observed in the air‐stability test, is noticeable after exposing the devices to operational conditions, suggesting that other external factors, such as photo‐ or heat‐induced degradation, are more responsible for the reduction of the built‐in field within the devices than air‐stability (Figures S12 and S14).

We now turn to the discussion of the recombination processes in degraded PSCs. Figure 3d displays the intensity dependence of *V*_OC_ for the degraded PSCs with different CILs. This measurement provides useful insight into the role of trap‐assisted recombination under *V*_OC_ conditions. A slope of unity *kT*/*q* (where *k* is the Boltzmann constant, and *T* and *q* represent the absolute temperature and elementary charge, respectively) is indicative of minimal recombination, whereas the stronger dependence of *V*_OC_ on the light intensity reflects the presence of the trap‐assisted recombination as primary loss mechanism. In the PSCs with significant degradation, trap‐assisted recombination prevails and the stable device with TiO_*x*_ CIL retains its nearly ideal recombination behavior (Figure S15). The rate of trap‐assisted recombination is closely related to the density of trap‐states. The dark *J*−*V* characteristics of electron‐only devices with a structure of FTO/SnO_2_/MAPbI_3_/PCBM/CIL/Cu were measured to derive trap density (*N*_t_) by using Equation 2:

(2) $$V_{TFL}=\frac{qN_{t}L^{2}}{2{\epsilon \epsilon }_{0}}$$

where *V*_TFL_ is the trap filling limit voltage and *L* stands for the thickness of the perovskite films (Figure 3e).^[64]^

The electron‐only devices were exposed to the same conditions as conducted in the operational stability test for PSCs and the calculated trap density *N*_t_ values in perovskite layers are summarized in Table S1. The results are well correlated with the trend observed in the recombination kinetics, indicating that CIL plays an important role in suppressing the evolution of trap states. The increased ionic defects may also influence the surface energy level of CILs. The surface energetics of the CILs on top of perovskite/PCBM were investigated by using a Kelvin probe method, resulting in changes in work functions of CILs upon degradation (Figure S16). This might be attributed to the fact that ionic defects are migrated and accumulated at the PCBM/CIL interfaces, affecting the effective work function of the CILs.^[65]^ In addition to the density, intra‐bandgap distribution of deep‐trap has an impact on the charge transport and recombination processes as charges become increasingly trapped in localized states. The characteristic energies (*E*_ch_) are obtained from the linear slope of the *J*−*V* characteristics of electron‐only devices, with smaller *E*_ch_ indicating a narrow deep‐trap distribution (Figure 3f).[^66^, ^67^] It has also been suggested that the trap states distribution exponentially decays towards the bandgap. Assuming that distribution of trap lying below the conduction band edge levels can be expressed by Equation 3:

(3) $$N\left(E-E_{CB}\right)=\frac{N_{t}}{E_{ch}}e^{\frac{E-E_{CB}}{E_{ch}}}$$

where *E*_CB_ is the energy of the conduction band edge. Considering the energetic location of Fermi level 0.3 eV below the conduction band level in MAPbI_3_ perovskite,^[68]^ increase in *E*_ch_ upon PSC degradation represent more charges are localized in the deep‐trap below the Fermi level of the perovskite (Figure S17 and Table S2).

**Figure 4:**
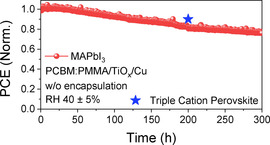
Continuous maximum power point (MPP) tracking for 300 h of non‐encapsulated MAPbI_3_ PSC under continuous standard illumination in an ambient atmosphere. The blue star indicates the normalized PCE value of the triple cation PSC after 200 h of stability test.

Considering the function of CILs as an inner encapsulation layer, it is expected that thicker CIL might be beneficial for enhancing device stability. To validate our assumption, we fabricated PSCs with a thicker TiO_*x*_ CIL layer and explored their device stability. By incorporating 1 wt % of poly(methyl methacrylate) (PMMA) into the PCBM layer and increasing the thickness of the TiO_*x*_ layer, the stability of the devices under operational condition can be improved, retaining >77 % of its initial PCE of 17.24 % for up to 300 h (Figure 4). Owing to the enhanced electrical conductivity of the codoped TiO_*x*_ layer, high device performance was observed, exhibiting an initial PCE of 17.24 % (Figure S18). It is worth noting that the perovskite composition used in this study is MAPbI_3_, which is known as a less stable composition. By introducing a stable triple cation perovskite system, the PSCs with Cs_0.05_(FA_0.83_MA_0.17_)Pb(I_0.83_Br_0.17_)_3_ exhibited prolonged device lifetime (Figure S19). With the newly developed doping concept and perovskite composition, the device durability could be further enhanced.

## Conclusion

In summary, we performed an in‐depth comparative study addressing the impact of CILs on the device stability in inverted p‐i‐n PSCs by using four different CILs (PFN, ZnO, BCP, and TiO_*x*_). In particular, a codoping strategy effectively enhanced the electrical properties of the TiO_*x*_ layer, which helps to improve not only the efficiency but also the stability of the devices. The degradation behaviors of PSCs were varied depending on the type of CILs, which is mainly attributed to the differences in performance between various CILs as barrier layers, despite their outstanding charge‐selective properties. Through comprehensive analysis of degraded PSC samples, increased internal resistance and energetic disorder were identified as critical factors of the degradation process, which are effectively suppressed with the introduction of the TiO_*x*_ layer. Finally, by incorporating a chemically modified TiO_*x*_ CIL, we significantly improved the operational stability of the PSCs, which retained over 77 % of their initial PCEs for up to 300 h. Our results provide useful insight into the PSC degradation mechanism and address the importance of the development of CILs that simultaneously satisfy both electrical and physical properties for achieving highly efficient and stable devices.

## Experimental Section

### Material preparation

Lead (II) iodide (PbI_2_, 99.99 %, trace metals basis) and methylamine hydroiodide (CH_3_NH_3_I, Low water content) were purchased from Tokyo Chemical Industry (TCI) and used without further purification. A 1.5 m perovskite precursor solution was prepared by dissolving PbI_2_ and CH_3_NH_3_I at a molar ratio of 1 : 1 in N,N‐dimethylformamide and dimethyl sulfoxide mixed solution (9 : 1.1 volume ratio) at 60 °C overnight. Titanium (IV) isopropoxide (TTIP), 3‐phenyl‐1‐propylamine (PPA), bis(diphenylphosphino)methane (DPPM), and bathocuproine (BCP, 96 %) and were purchased from Sigma‐Aldrich. The BCP solution was prepared by dissolving BCP in methanol with a concentration of 0.05 wt %. The poly(bis(4‐phenyl)(2,4,6‐trimethylphenyl)amine) (PTAA) and poly(9,9‐bis(3′‐(*N*,*N*‐dimethyl)‐*N*‐ethylammonium‐propyl‐2,7‐fluorene)‐*alt*‐2,7‐(9,9‐dioctylfluorene))dibromide (PFN−Br) were purchased from 1‐Material and dissolved in toluene (anhydrous, 99.8 %) and methanol (anhydrous, 99.8 %) at a concentration of 2 mg/mL and 0.5 mg/mL, respectively. All other materials and solvents were purchased from Sigma‐Aldrich and used without further purification unless otherwise stated.

#### Synthesis of ZnO nanoparticles

The ZnO nanoparticles were prepared using a modified literature procedure.^[69]^ Briefly, zinc acetate (164 mg; ACS reagent, ≥98 %), methanol (8.4 mL), and deionized water (50 μL) were added to a solution of potassium hydroxide (88 mg) in methanol (4.6 mL) at 60 °C under magnetic stirring. After 4 h of reacting process, the precipitate was washed and collected by centrifugation. The synthesized ZnO nanoparticles were then diluted to 1 wt % in isopropanol (IPA). To obtain a well‐dispersed ZnO nanoparticle colloidal solution, ethanolamine (0.2 wt %) was further added, which was sonicated for 30 min.

#### Synthesis of TiO_*x*_ precursor solution

TTIP (300 μL) and PPA (600 μL) were added dropwise into 2‐methoxyethanol (3 mL) and stirred for 1 h. The transparent solution was then heated at reflux for 4 h at 150 °C under vigorous stirring to yield a reddish yellow solution. 1 mL of the resulting amine‐mediated TiO_*x*_ solution was mixed with DPPM (600 mg), toluene (10 mL) and IPA (20 mL). All synthetic processes were conducted in an argon atmosphere to prevent hydrolysis.

### Device fabrication

The fluorine doped tin oxide coated (FTO) glass (surface resistance ≈7 Ω square^−1^) was etched using Zn powder and etching solution that was prepared by mixing 2.0 m HCl with DI water with a volume ratio of 1 : 1. First, zinc powder is applied to an area to be etched, and then an etching solution is dropped. After finishing the etching process, the etched FTO substrate was ultrasonically washed with deionized water, acetone, and IPA each for 15 min. Finally, the cleaned FTO substrate was treated with UV‐ozone for 20 min. The UV‐ozone treated substrates were then moved into the homemade dry‐box filled with argon (Ar) gas. Note that all of the device fabrication processes have been done under the Ar atmosphere except the UV‐ozone treatment. For the fabrication of the inverted PSCs, the PTAA solution was spin‐coated onto the FTO substrate at 3000 rpm for the 30 s, then annealed on hot plate at 60 °C for 10 min. Subsequently, PFN solution was spin‐coated onto the PTAA at 5000 rpm. The polycrystalline perovskite film was obtained by spin‐coating perovskite precursor solution at 3000 rpm for 30 s and subsequently dripping of diethyl ether (1 mL) after 12 s. The samples were then annealed on a hot plate at 100 °C for 10 min. The PC_61_BM solution (3 wt % in chlorobenzene) was spin‐coated onto the perovskite layers at 2000 rpm for 30 s, then annealed at 100 °C for 5 min. All CILs (e. g., PFN, ZnO, BCP, and TiO_*x*_) were deposited under same conditions: spin‐coated at 5000 rpm for 30 s. The PSC device fabrication was completed by thermal evaporation of Cu electrode (100 nm) through shadow masks under high vacuum (1×10^−6^ torr) using a thermal evaporator (Kurt J. Lesker) placed in an ambient environment. For the triple cation PSC fabrication, formamidinium iodide (FAI; 172 mg), PbI_2_ (461 mg), methylammonium bromide (MABr, 22.4 mg), PbBr_2_ (80.7 mg) were dissolved in 4 : 1 v/v DMF/DMSO (1 mL). The diethyl ether‐treated triple cation perovskite samples were annealed on a hot plate at 100 C for 1 h. In the case of electron‐only device fabrication (FTO/SnO_2_/perovskite/PCBM/CIL/Cu), except for the SnO_2_ deposition, all layers were deposited under the same process conditions as those used in the PSC. The SnO_2_ layer was deposited by spin‐coating a precursor solution containing tin(II) chloride dehydrate (65 mg; ≥99.995 %) and IPA (3 mL) and was annealed 185 °C for 1 h.

### Characterizations of films and devices

The UV/Vis absorption spectra were measured by using a spectrophotometer (Lambda 365 UV/Vis spectrometer, PerkinElmer). Fourier transform infrared (FTIR) spectra were recorded by a Spectrum TwoTM FTIR spectrometer (PerkinElmer). The steady‐state photoluminescence (PL) measurement was performed using a spectrometer (FS5 spectrofluorometer, Edinburgh Instruments). PL spectra of the perovskite films were obtained by scanning the emission monochromator from 650 to 850 nm under light source excitation at a fixed wavelength of 450 nm. *J*−*V* characteristics were recorded on the source meter unit (Keithley 2450 Graphical SourceMeter SMU Instruments, Tektronix), where a solar simulator (Oriel LCS‐100 lamp with an AM 1.5G filter, Newport, 100 mW cm^−2^) was used for photovoltaic measurements. The operational stability of PSC was monitored at maximum power point (MPP) tracking under continuous illumination, in which the same light source was used when measuring the *J*−*V* characteristics. For air stability test, PSC devices were stored in an ambient condition without encapsulation and performance was measured periodically.

For the electrochemical impedance spectroscopy (EIS) measurement of the devices, the frequency response analyzer (FRA) system of the Biologic SP‐150 potentiostat was used. The EIS spectra were collected over the frequency range of 1 MHz‐1 Hz at different applied bias values (depending on the CILs) under dark condition. The capacitance‐voltage (*C*−*V*) characteristics of PSCs for the Mott‐Schottky plot were measured under one sun illumination and the frequency of 100 kHz at bias potentials between −1.0 and 1.5 V by using the same system as in the EIS measurement.

X‐ray photoelectron spectroscopy (XPS) measurements were performed using a SPECS GmbH spectrometer with a PHOIBOS 150 1D‐DLD hemispherical analyzer and a monochromatized Al_Kα_ X‐ray source. The core level XPS spectra were recorded using a pass energy of 20 eV. The binding energy was calibrated by the peak at 284.5 eV of C 1s spectra. The medium area mode was used as lens mode. The base pressure during the experiment in the analysis chamber was 5×10^−10^ mbar. The contact potential difference of each sample was recorded by a Kelvin probe (KP 6500 Digital Kelvin probe, McAllister Technical Services. Co. Ltd) and calibrated to highly ordered pyrolytic graphite (HOPG) at 4.55±0.05 eV.

X‐ray diffraction (XRD) patterns were collected on Stoe STADI P transmission diffractometer equipped with a primary Ge (111) monochromator (Mo_Kα1_) and a position‐sensitive detector. Data were collected in the 2*θ* range between 5 and 90° with a step width of 0.05° and a measuring time per step of 30 s.

Scanning transmission electron microscopy (STEM) and energy dispersive X‐ray spectrometry (EDS) elemental mapping were recorded on a Hitachi HD‐2700 C_s_‐corrected STEM equipped with cold field‐emission gun (FEG) and EDAX Octane T Ultra W 200 mm^2^ silicon drift detector (SDD) and operated at 200 kV. The analysis of the data was supported by EDAX Team software. Scanning electron microscopy (SEM) images were taken with Hitachi S‐5500 microscopy.

## Conflict of interest

The authors declare no conflict of interest.

## Supporting information

As a service to our authors and readers, this journal provides supporting information supplied by the authors. Such materials are peer reviewed and may be re‐organized for online delivery, but are not copy‐edited or typeset. Technical support issues arising from supporting information (other than missing files) should be addressed to the authors.

Supplementary

## References

[1] M. Kaltenbrunner , G. Adam , E. D. Głowacki , M. Drack , R. Schwödiauer , L. Leonat , D. H. Apaydin , H. Groiss , M. C. Scharber , M. S. White , N. S. Sariciftci , S. Bauer , Nat. Mater. 2015, 14, 1032–1039. 26301766 10.1038/nmat4388

[2] S. Kang , J. Jeong , S. Cho , Y. J. Yoon , S. Park , S. Lim , J. Y. Kim , H. Ko , J. Mater. Chem. A 2019, 7, 1107–1114.

[3] A. Miyata , A. Mitioglu , P. Plochocka , O. Portugall , J. T. W. Wang , S. D. Stranks , H. J. Snaith , R. J. Nicholas , Nat. Phys. 2015, 11, 582–587.

[4] Q. Lin , A. Armin , R. C. R. Nagiri , P. L. Burn , P. Meredith , Nat. Photonics 2015, 9, 106–112.

[5] C. Wehrenfennig , G. E. Eperon , M. B. Johnston , H. J. Snaith , L. M. Herz , Adv. Mater. 2014, 26, 1584–1589. 24757716 10.1002/adma.201305172 PMC4722848

[6] T. J. Savenije , C. S. Ponseca , L. Kunneman , M. Abdellah , K. Zheng , Y. Tian , Q. Zhu , S. E. Canton , I. G. Scheblykin , T. Pullerits , A. Yartsev , V. Sundström , J. Phys. Chem. Lett. 2014, 5, 2189–2194. 26279532 10.1021/jz500858a

[7] Y. Yang , M. Yang , D. Moore , Y. Yan , E. Miller , K. Zhu , M. Beard , Nat. Energy 2017, 2, 16207.

[8] S. D. Stranks , G. E. Eperon , G. Grancini , C. Menelaou , M. J. P. Alcocer , T. Leijtens , L. M. Herz , A. Petrozza , H. J. Snaith , Science 2013, 342, 341–344. 24136964 10.1126/science.1243982

[9] G. Xing , N. Mathews , S. Sun , S. S. Lim , Y. M. Lam , M. Grätzel , S. Mhaisalkar , T. C. Sum , Science 2013, 342, 344–347. 24136965 10.1126/science.1243167

[10] V. Gonzalez-Pedro , E. J. Juarez-Perez , W. S. Arsyad , E. M. Barea , F. Fabregat-Santiago , I. Mora-Sero , J. Bisquert , Nano Lett. 2014, 14, 888–893. 24397375 10.1021/nl404252e

[11] C. W. Chen , S. Y. Hsiao , C. Y. Chen , H. W. Kang , Z. Y. Huang , H. W. Lin , J. Mater. Chem. A 2015, 3, 9152–9159.

[12] G. E. Eperon , S. D. Stranks , C. Menelaou , M. B. Johnston , L. M. Herz , H. J. Snaith , Energy Environ. Sci. 2014, 7, 982–988.

[13] L. Wang , G. D. Yuan , R. F. Duan , F. Huang , T. B. Wei , Z. Q. Liu , J. X. Wang , J. M. Li , AIP Adv. 2016, 6, 045115.

[14] D. P. McMeekin , G. Sadoughi , W. Rehman , G. E. Eperon , M. Saliba , M. T. Hörantner , A. Haghighirad , N. Sakai , L. Korte , B. Rech , M. B. Johnston , L. M. Herz , H. J. Snaith , Science 2016, 351, 151–155. 26744401 10.1126/science.aad5845

[15] S. Schünemann , K. Chen , S. Brittman , E. Garnett , H. Tüysüz , ACS Appl. Mater. Interfaces 2016, 8, 25489–25495. 27589559 10.1021/acsami.6b09227

[16] K. Chen , S. Schünemann , S. Song , H. Tüysüz , Chem. Soc. Rev. 2018, 47, 7045–7077. 30101254 10.1039/c8cs00212f

[17] Y. Dai , C. Poidevin , C. Ochoa-Hernández , A. A. Auer , H. Tüysüz , Angew. Chem. Int. Ed. 2020, 59, 5788–5796; 10.1002/anie.201915034 PMC7154683 31850662

[18] C. C. Stoumpos , C. D. Malliakas , M. G. Kanatzidis , Inorg. Chem. 2013, 52, 9019–9038. 23834108 10.1021/ic401215x

[19] S. Shrestha , R. Fischer , G. J. Matt , P. Feldner , T. Michel , A. Osvet , I. Levchuk , B. Merle , S. Golkar , H. Chen , S. F. Tedde , O. Schmidt , R. Hock , M. Rührig , M. Göken , W. Heiss , G. Anton , C. J. Brabec , Nat. Photonics 2017, 11, 436–440.

[20] Best Research-Cell Efficiency Chart from National Renewable Energy Laboratory (NREL), https://www.nrel.gov/pv/cell-efficiency.html (accessed: January 2021).

[21] G. Niu , X. Guo , L. Wang , J. Mater. Chem. A 2015, 3, 8970–8980.

[22] D. Wang , M. Wright , N. K. Elumalai , A. Uddin , Sol. Energy Mater. Sol. Cells 2016, 147, 255–275.

[23] N. G. Park , M. Grätzel , T. Miyasaka , K. Zhu , K. Emery , Nat. Energy 2016, 1, 16152–16159.

[24] G. Grancini , C. Roldán-Carmona , I. Zimmermann , E. Mosconi , X. Lee , D. Martineau , S. Narbey , F. Oswald , F. De Angelis , M. Graetzel , M. K. Nazeeruddin , Nat. Commun. 2017, 8, 15684. 28569749 10.1038/ncomms15684 PMC5461484

[25] P. Li , C. Liang , B. Bao , Y. Li , X. Hu , Y. Wang , Y. Zhang , F. Li , G. Shao , Y. Song , Nano Energy 2018, 46, 203–211.

[26] Y. Rong , Y. Hu , A. Mei , H. Tan , M. I. Saidaminov , S. Il Seok , M. D. McGehee , E. H. Sargent , H. Han , Science 2018, 361, eaat8235. 30237326 10.1126/science.aat8235

[27] Y. Chen , L. Zhang , Y. Zhang , H. Gao , H. Yan , RSC Adv. 2018, 8, 10489–10508. 10.1039/c8ra00384j PMC9078911 35540458

[28] T. Leijtens , G. E. Eperon , S. Pathak , A. Abate , M. M. Lee , H. J. Snaith , Nat. Commun. 2013, 4, 2885. 24301460 10.1038/ncomms3885

[29] A. K. Jena , H. W. Chen , A. Kogo , Y. Sanehira , M. Ikegami , T. Miyasaka , ACS Appl. Mater. Interfaces 2015, 7, 9817–9823. 25905438 10.1021/acsami.5b01789

[30] W. S. Yang , J. H. Noh , N. J. Jeon , Y. C. Kim , S. Ryu , J. Seo , S. Il Seok , Science 2015, 348, 1234–1237. 25999372 10.1126/science.aaa9272

[31] N. J. Jeon , H. Na , E. H. Jung , T. Y. Yang , Y. G. Lee , G. Kim , H. W. Shin , S. Il Seok , J. Lee , J. Seo , Nat. Energy 2018, 3, 682–689.

[32] E. H. Jung , N. J. Jeon , E. Y. Park , C. S. Moon , T. J. Shin , T. Y. Yang , J. H. Noh , J. Seo , Nature 2019, 567, 511–515. 30918371 10.1038/s41586-019-1036-3

[33] Q. Dong , Y. Shi , K. Wang , Y. Li , S. Wang , H. Zhang , Y. Xing , Y. Du , X. Bai , T. Ma , J. Phys. Chem. C 2015, 119, 10212–10217.

[34] Z. Zhu , Y. Bai , X. Liu , C. C. Chueh , S. Yang , A. K. Y. Jen , Adv. Mater. 2016, 28, 6478–6484. 27168338 10.1002/adma.201600619

[35] Q. Jiang , X. Zhang , J. You , Small 2018, 14, 1801154.

[36] Y. Shao , Y. Yuan , J. Huang , Nat. Energy 2016, 1, 16039.

[37] L. Meng , J. You , T. F. Guo , Y. Yang , Acc. Chem. Res. 2016, 49, 155–165. 26693663 10.1021/acs.accounts.5b00404

[38] W. Yan , S. Ye , Y. Li , W. Sun , H. Rao , Z. Liu , Z. Bian , C. Huang , Adv. Energy Mater. 2016, 6, 1600474.

[39] Z. Liu , A. Zhu , F. Cai , L. M. Tao , Y. Zhou , Z. Zhao , Q. Chen , Y. B. Cheng , H. Zhou , J. Mater. Chem. A 2017, 5, 6597–6605.

[40] C. C. Chueh , C. Z. Li , A. K. Y. Jen , Energy Environ. Sci. 2015, 8, 1160–1189.

[41] Z. Zhu , Y. Bai , X. Liu , C.-C. Chueh , S. Yang , A. K.-Y. Jen , Adv. Mater. 2016, 28, 6478–6484. 27168338 10.1002/adma.201600619

[42] X. Lin , D. Cui , X. Luo , C. Zhang , Q. Han , Y. Wang , L. Han , Energy Environ. Sci. 2020, 13, 3823–3847.

[43] I. C. Smith , E. T. Hoke , D. Solis-Ibarra , M. D. McGehee , H. I. Karunadasa , Angew. Chem. Int. Ed. 2014, 53, 11232–11235; 10.1002/anie.201406466 25196933

[44] I. Hwang , I. Jeong , J. Lee , M. J. Ko , K. Yong , ACS Appl. Mater. Interfaces 2015, 7, 17330–17336. 26154828 10.1021/acsami.5b04490

[45] Q. Wang , B. Chen , Y. Liu , Y. Deng , Y. Bai , Q. Dong , J. Huang , Energy Environ. Sci. 2017, 10, 516–522.

[46] J. Jiang , Q. Wang , Z. Jin , X. Zhang , J. Lei , H. Bin , Z. G. Zhang , Y. Li , S. F. Liu , Adv. Energy Mater. 2018, 8, 1701757.

[47] W. Li , H. Dong , L. Wang , N. Li , X. Guo , J. Li , Y. Qiu , J. Mater. Chem. A 2014, 2, 13587–13592.

[48] A. Guerrero , J. You , C. Aranda , Y. S. Kang , G. Garcia-Belmonte , H. Zhou , J. Bisquert , Y. Yang , ACS Nano 2016, 10, 218–224. 26679510 10.1021/acsnano.5b03687

[49] H. Back , G. Kim , J. Kim , J. Kong , T. K. Kim , H. Kang , H. Kim , J. Lee , S. Lee , K. Lee , Energy Environ. Sci. 2016, 9, 1258–1263.

[50] S. Wu , R. Chen , S. Zhang , B. H. Babu , Y. Yue , H. Zhu , Z. Yang , C. Chen , W. Chen , Y. Huang , S. Fang , T. Liu , L. Han , W. Chen , Nat. Commun. 2019, 10, 1161. 30858370 10.1038/s41467-019-09167-0 PMC6411982

[51] E. M. Sanehira , B. J. Tremolet De Villers , P. Schulz , M. O. Reese , S. Ferrere , K. Zhu , L. Y. Lin , J. J. Berry , J. M. Luther , ACS Energy Lett. 2016, 1, 38–45.

[52] Y. Hou , X. Du , S. Scheiner , D. P. McMeekin , Z. Wang , N. Li , M. S. Killian , H. Chen , M. Richter , I. Levchuk , N. Schrenker , E. Spiecker , T. Stubhan , N. A. Luechinger , A. Hirsch , P. Schmuki , H. P. Steinrück , R. H. Fink , M. Halik , H. J. Snaith , C. J. Brabec , Science 2017, 358, 1192–1197. 29123021 10.1126/science.aao5561

[53] N. Arora , M. I. Dar , A. Hinderhofer , N. Pellet , F. Schreiber , S. M. Zakeeruddin , M. Grätzel , Science 2017, 358, 768–771. 28971968 10.1126/science.aam5655

[54] J. A. Christians , P. Schulz , J. S. Tinkham , T. H. Schloemer , S. P. Harvey , B. J. Tremolet De Villers , A. Sellinger , J. J. Berry , J. M. Luther , Nat. Energy 2018, 3, 68–74.

[55] K. O. Brinkmann , J. Zhao , N. Pourdavoud , T. Becker , T. Hu , S. Olthof , K. Meerholz , L. Hoffmann , T. Gahlmann , R. Heiderhoff , M. F. Oszajca , N. A. Luechinger , D. Rogalla , Y. Chen , B. Cheng , T. Riedl , Nat. Commun. 2017, 8, 15341. 28067308 10.1038/ncomms13938 PMC5336555

[56] C. C. Boyd , R. Cheacharoen , K. A. Bush , R. Prasanna , T. Leijtens , M. D. McGehee , ACS Energy Lett. 2018, 3, 1772–1778.

[57] S. Seo , S. Jeong , C. Bae , N. G. Park , H. Shin , Adv. Mater. 2018, 30, 1801010. 10.1002/adma.201801010 29786887

[58] G. Kim , J. Kong , J. Kim , H. Kang , H. Back , H. Kim , K. Lee , Adv. Energy Mater. 2015, 5, 1401298.

[59] H. Back , G. Kim , H. Kim , C. Y. Nam , J. Kim , Y. R. Kim , T. Kim , B. Park , J. R. Durrant , K. Lee , Energy Environ. Sci. 2020, 13, 840–847.

[60] W. Chen , Y. Z. Wu , Y. F. Yue , J. Liu , W. J. Zhang , X. D. Yang , H. Chen , E. B. Bi , I. Ashraful , M. Grätzel , L. Y. Han , Science 2015, 350, 944–948. 26516198 10.1126/science.aad1015

[61] H. Tan , A. Jain , O. Voznyy , X. Lan , F. P. García de Arquer , J. Z. Fan , R. Quintero-Bermudez , M. Yuan , B. Zhang , Y. Zhao , F. Fan , P. Li , L. N. Quan , Y. Zhao , Z.-H. Lu , Z. Yang , S. Hoogland , E. H. Sargent , Science 2017, 355, 722–726. 28154242 10.1126/science.aai9081

[62] J. Kong , J. Lee , G. Kim , H. Kang , Y. Choi , K. Lee , Phys. Chem. Chem. Phys. 2012, 14, 10547–10555. 22739643 10.1039/c2cp41501a

[63] W. Muhammad , N. Ullah , M. Haroon , B. H. Abbasi , RSC Adv. 2019, 9, 29541–29548. 10.1039/c9ra04424h PMC9071912 35531532

[64] A. Poglitsch , D. Weber , J. Chem. Phys. 1987, 87, 6373–6378.

[65] A. Guerrero , J. You , C. Aranda , Y. S. Kang , G. Garcia-Belmonte , H. Zhou , J. Bisquert , Y. Yang , ACS Nano 2016, 10, 218–224. 26679510 10.1021/acsnano.5b03687

[66] M. M. Mandoc , B. de Boer , G. Paasch , P. W. M. Blom , Phys. Rev. B 2007, 75, 193202.

[67] J. Wu , J. Lee , Y. C. Chin , H. Yao , H. Cha , J. Luke , J. Hou , J. S. Kim , J. R. Durrant , Energy Environ. Sci. 2020, 13, 2422–2430.

[68] P. Schulz , E. Edri , S. Kirmayer , G. Hodes , D. Cahen , A. Kahn , Energy Environ. Sci. 2014, 7, 1377–1381.

[69] S. Ben Dkhil , D. Duché , M. Gaceur , A. K. Thakur , F. B. Aboura , L. Escoubas , J. J. Simon , A. Guerrero , J. Bisquert , G. Garcia-Belmonte , Q. Bao , M. Fahlman , C. Videlot-Ackermann , O. Margeat , J. Ackermann , Adv. Energy Mater. 2014, 4, 1400805.

