# Supplementary material for: In‐Depth Comparative Study of the Cathode Interfacial Layer for a Stable Inverted Perovskite Solar Cell
Source: ChemSusChem. 2021 May 7;14(11):2393–400. doi: 10.1002/cssc.202100585 (PMC8251563; doi:10.1002/cssc.202100585)
Supplement: Supplementary file 1 — Supplementary [file CSSC-14-2393-s001.pdf]

ChemSusChem

Supporting Information

In-Depth Comparative Study of the Cathode Interfacial Layer for a Stable Inverted Perovskite Solar Cell

Jinho Lee and Harun Tüysüz*© 2021 The Authors. ChemSusChem published by Wiley-VCH GmbH. This is an open access article under the terms of the Creative Commons Attribution License, which permits use, distribution and reproduction in any medium, provided the original work is properly cited.

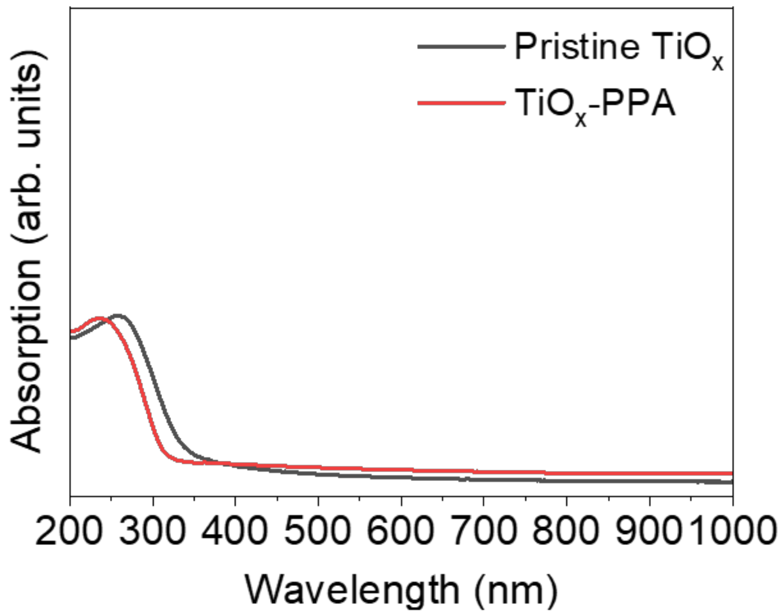

Figure S1. UV-vis absorption spectra of pristine TiO_x and TiO_x-PPA.

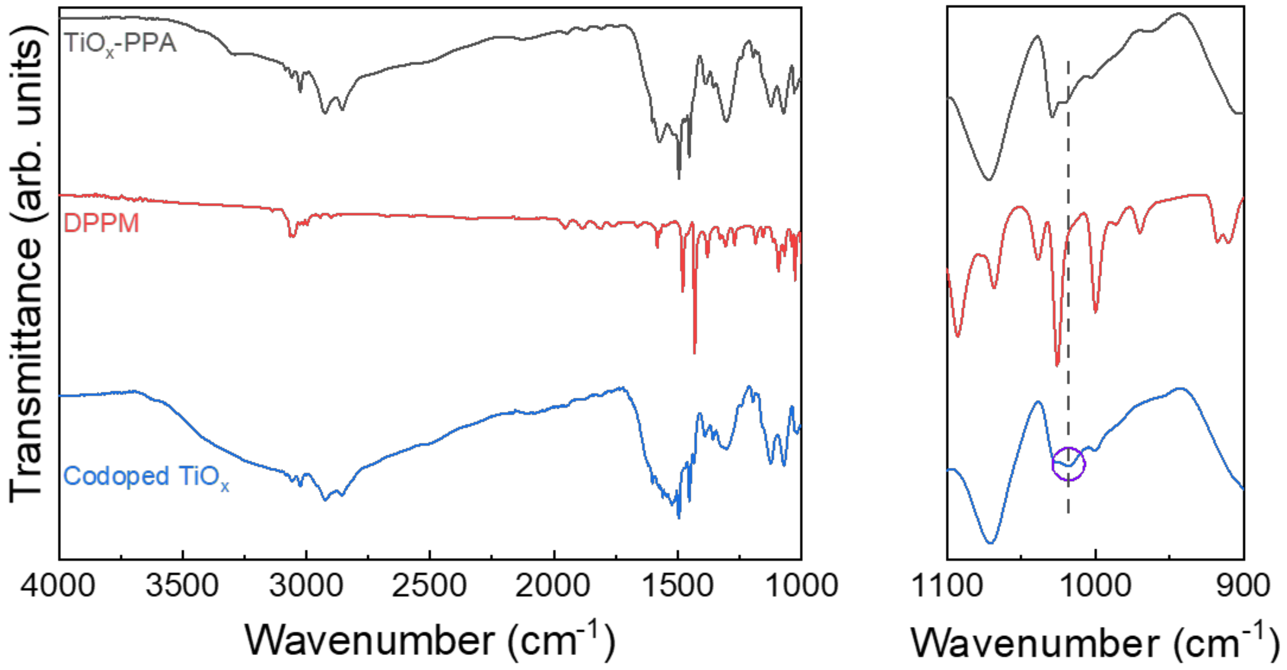

Figure S2. FTIR spectra and their magnified scanning region (right) of the TiO_x-PPA, DPPM, and codoped TiO_x. The marked violet circle in magnified spectra represents the evolution of new peak with the addition of DPPM in TiO_x-PPA matrix.

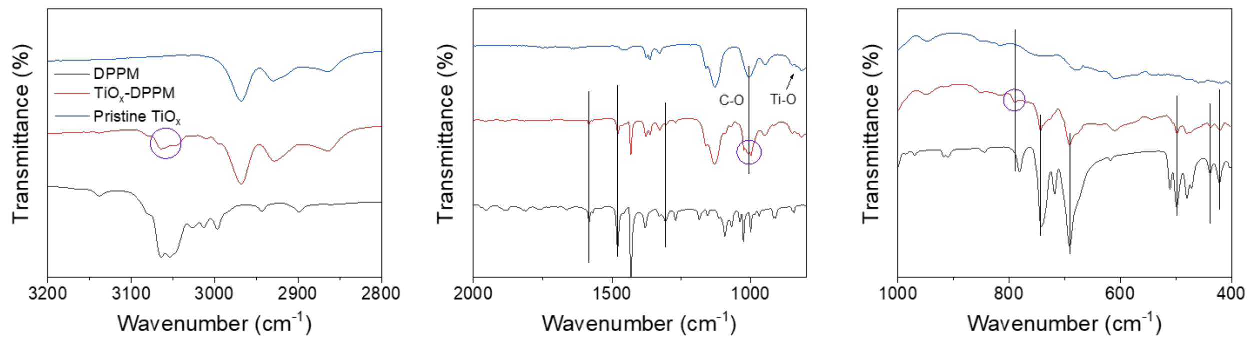

Figure S3. FTIR spectra of DPPM, pristine TiO_x, and TiO_x-DPPM films.

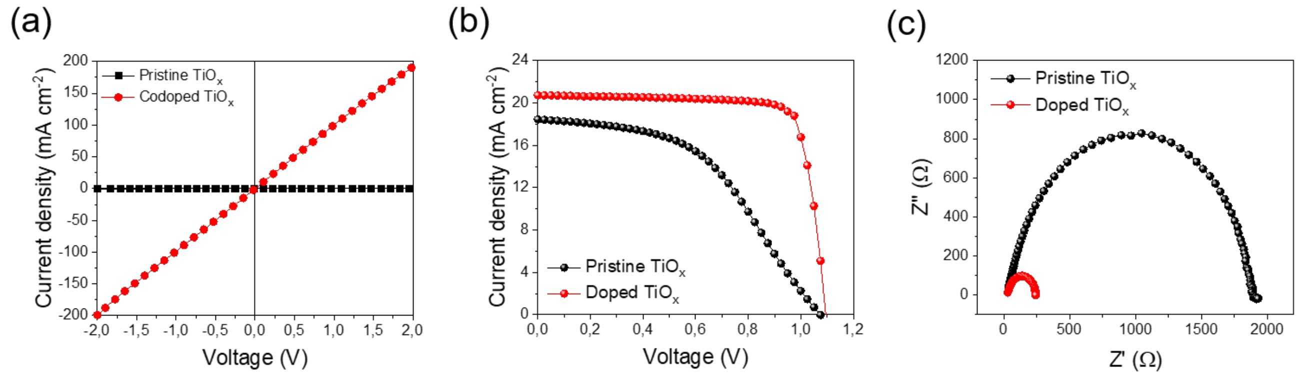

Figure S4. a) *J-V* characteristics of metal/pristine TiO_x or codoped TiO_x/metal devices. b) *J-V* characteristics and c) corresponding impedance spectra of PSCs with pristine TiO_x and codoped TiO_x.

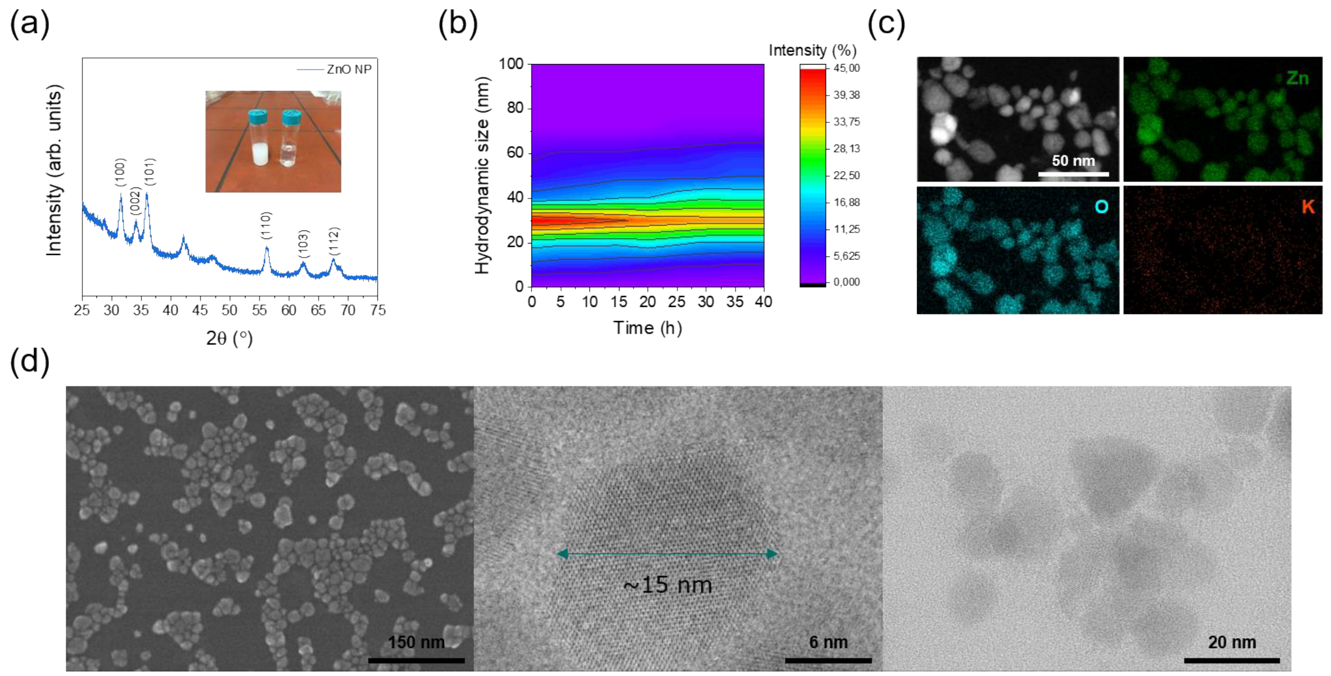

Figure S5. a) XRD pattern of synthesized ZnO nanoparticles. Inset displays a photograph of colloidal solutions before (left) and after (right) homogeneous dispersion. b) Size distribution of ZnO nanoparticle as a function of aging time, derived from dynamic light scattering (DLS) measurement results. c) Scanning transmission electron microscopy (STEM) and corresponding elemental mapping images of synthesized ZnO nanoparticles. d) HR-TEM images of ZnO nanoparticles.

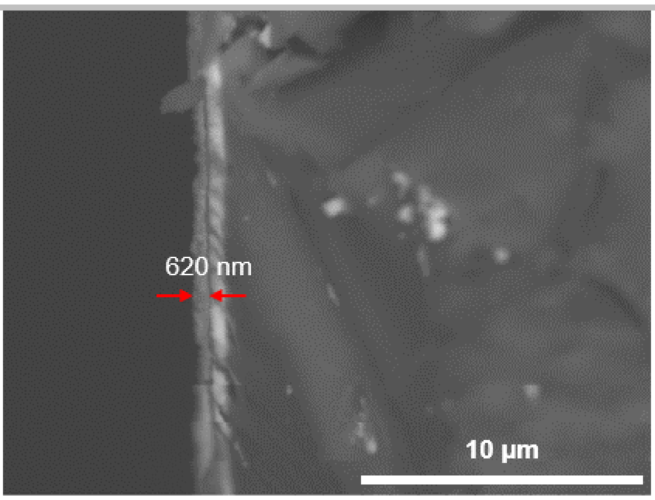

Figure S6. Cross-sectional SEM image for PSC device; approximately, 20 nm of PTAA, 400 nm of $MAPbI_3$, 80 nm of PCBM, and 20 nm of TiO_x, and 100 nm of Cu.

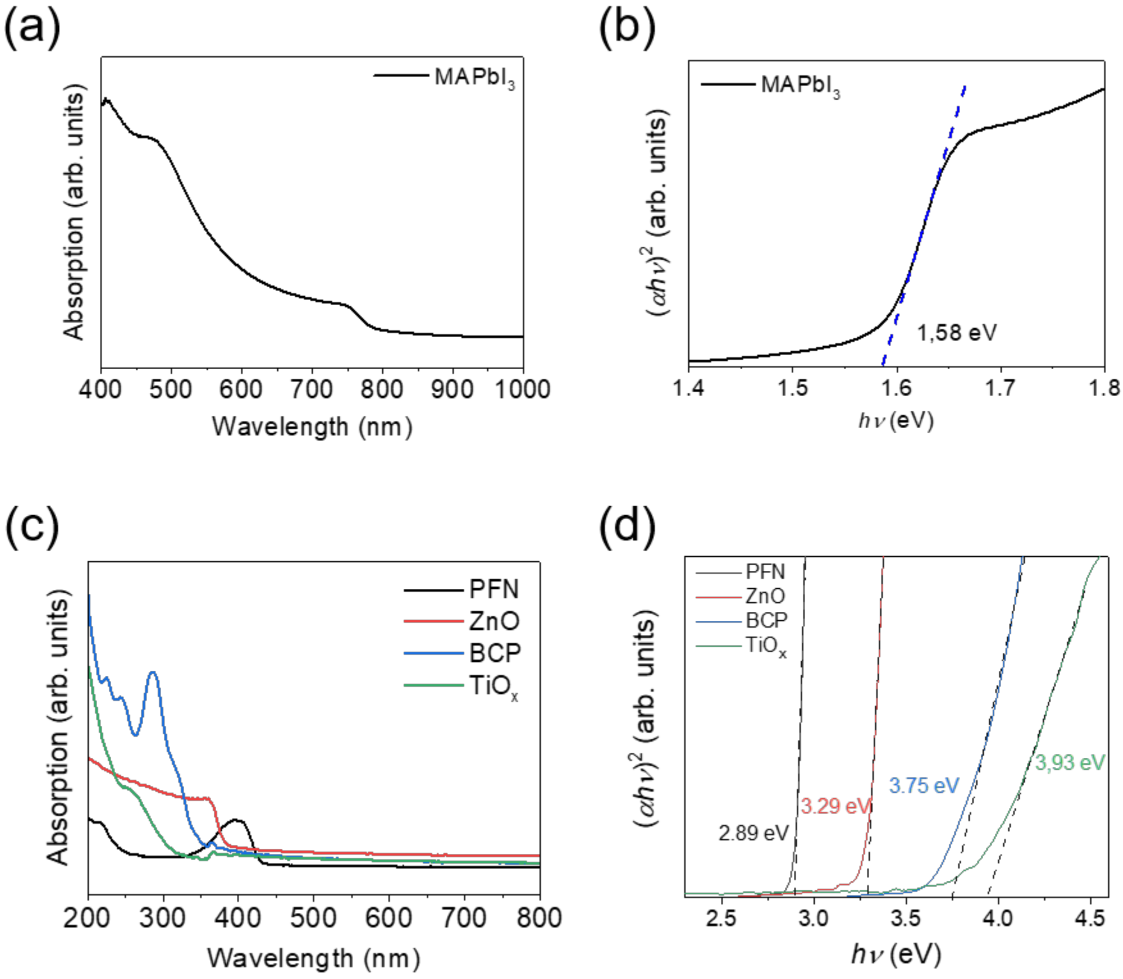

Figure S7. a) UV-vis absorption spectrum and b) Tauc plot of $MAPbI_3$ perovskite film. c) UV-vis absorption spectra and d) Tauc plots of CILs, including PFN, ZnO, BCP, and TiO_x thin films.

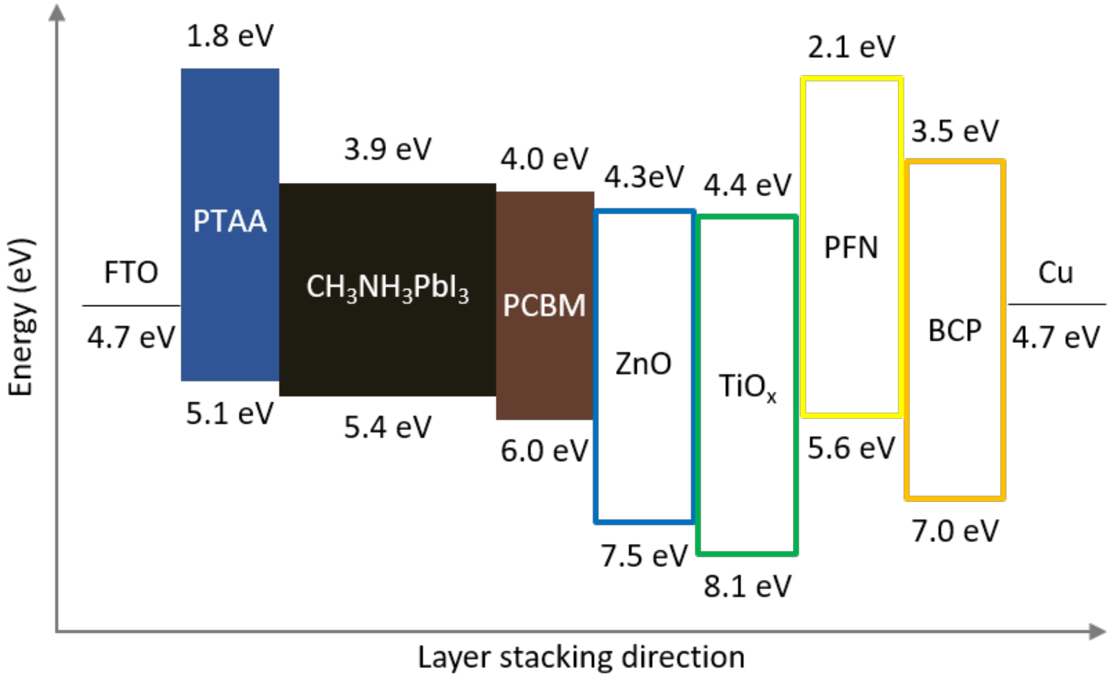

Figure S8. Energy level diagram of inverted p-i-n PSC device.

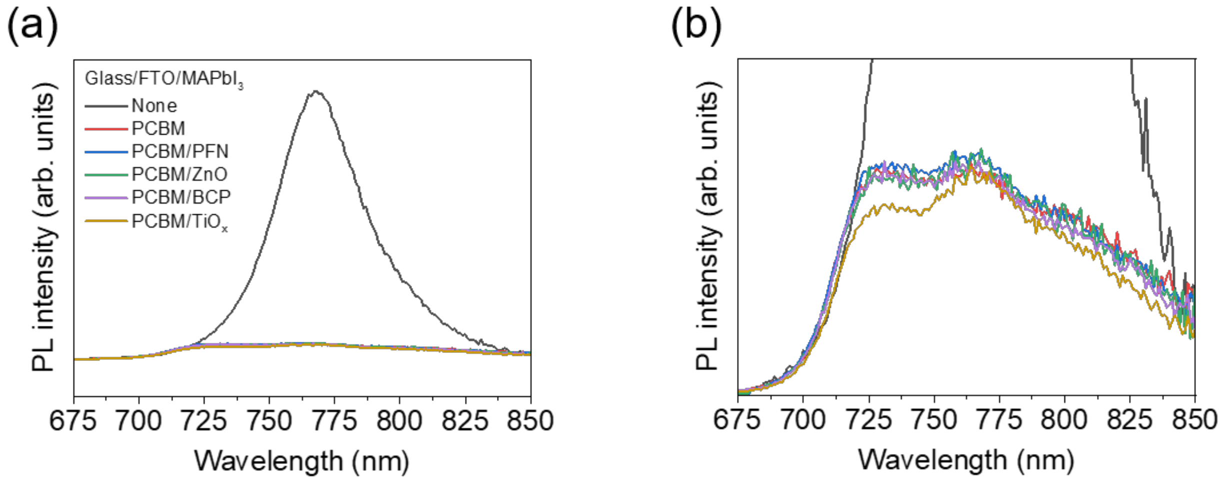

Figure S9. a) Photoluminescence (PL) spectra and b) their magnified scanning region of perovskite films deposited on the glass/FTO substrate with configurations of perovskite, perovskite/PCBM, and perovskite/PCBM/CIL.

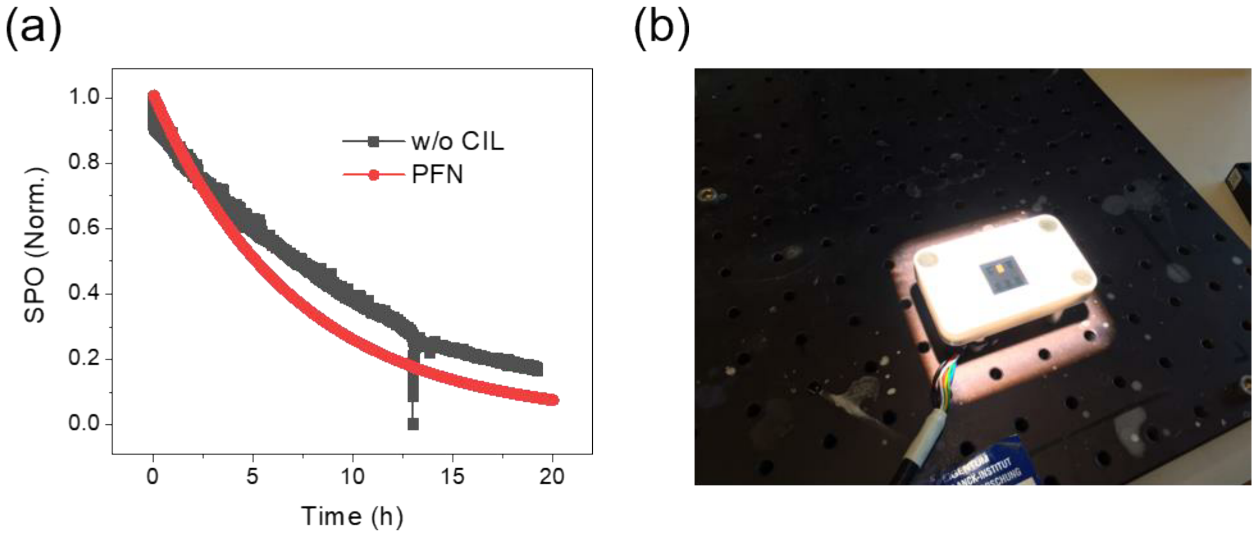

Figure S10. a) Performance degradation profiles of devices without CIL and with PFN CIL under continuous standard illumination in an ambient atmosphere. b) Photograph displaying the device without CIL during the stability test. The remarkable change in the color of specific area indicates significant decomposition of the perovskite within active area of the device.

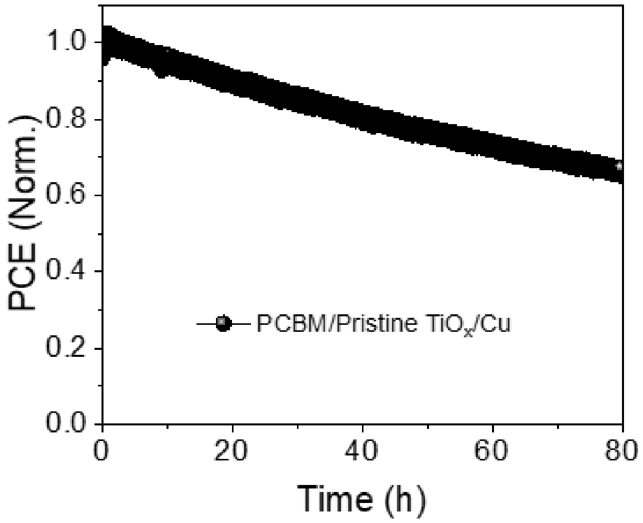

Figure S11. MPP tracking of PSC with pristine TiO_x CIL.

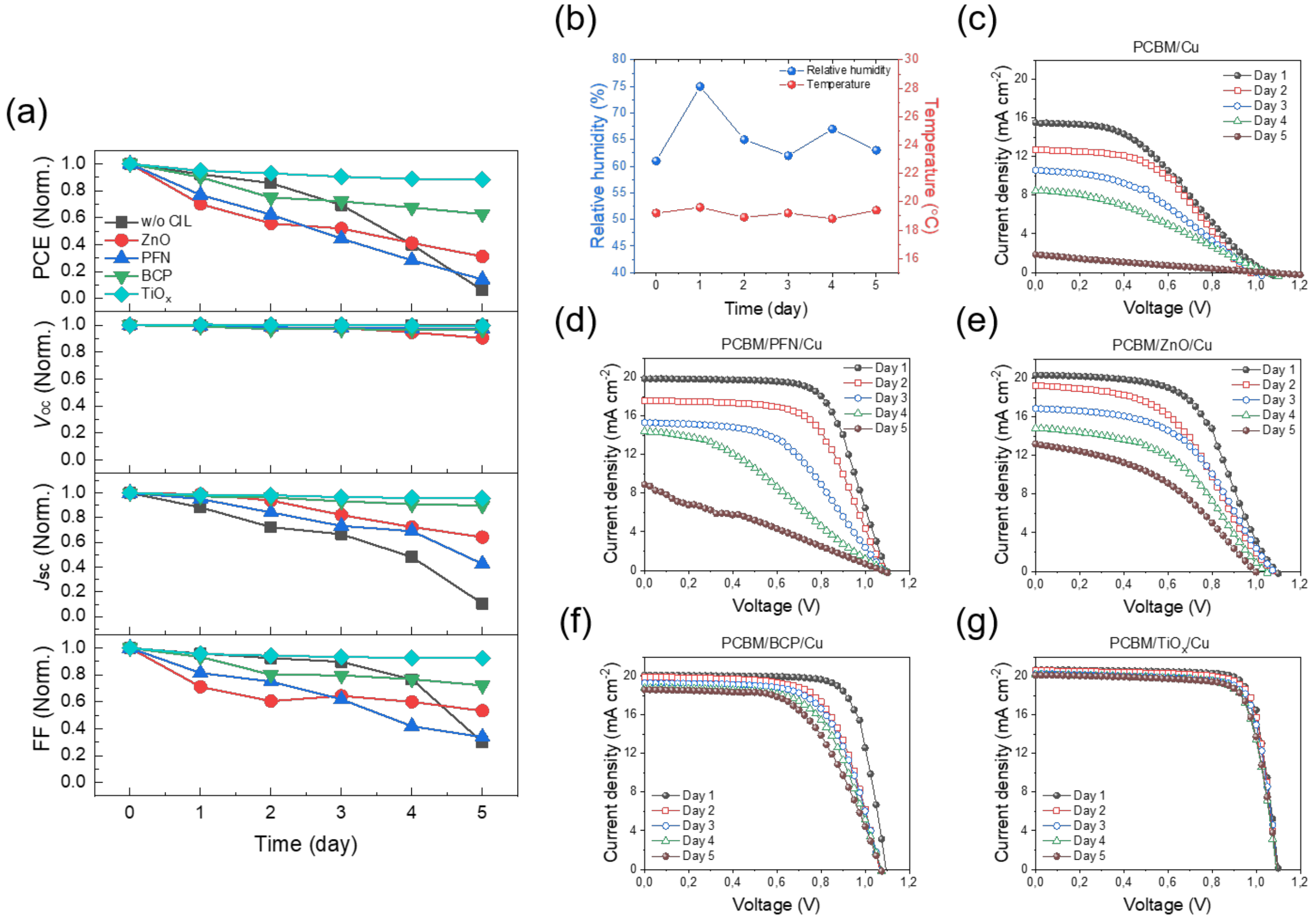

Figure S12. a) Normalized degradation profiles of photovoltaic parameters, including PCE, V_{OC}, J_{SC}, and FF, over time during storage in air without additional encapsulation. b) Recorded values for RH and temperature during air stability test. Changes in *J-V* characteristics of the PSCs c) without CIL, d) PFN, e) ZnO, f) BCP, and g) TiO_x, as a function of storage time in an ambient atmosphere.

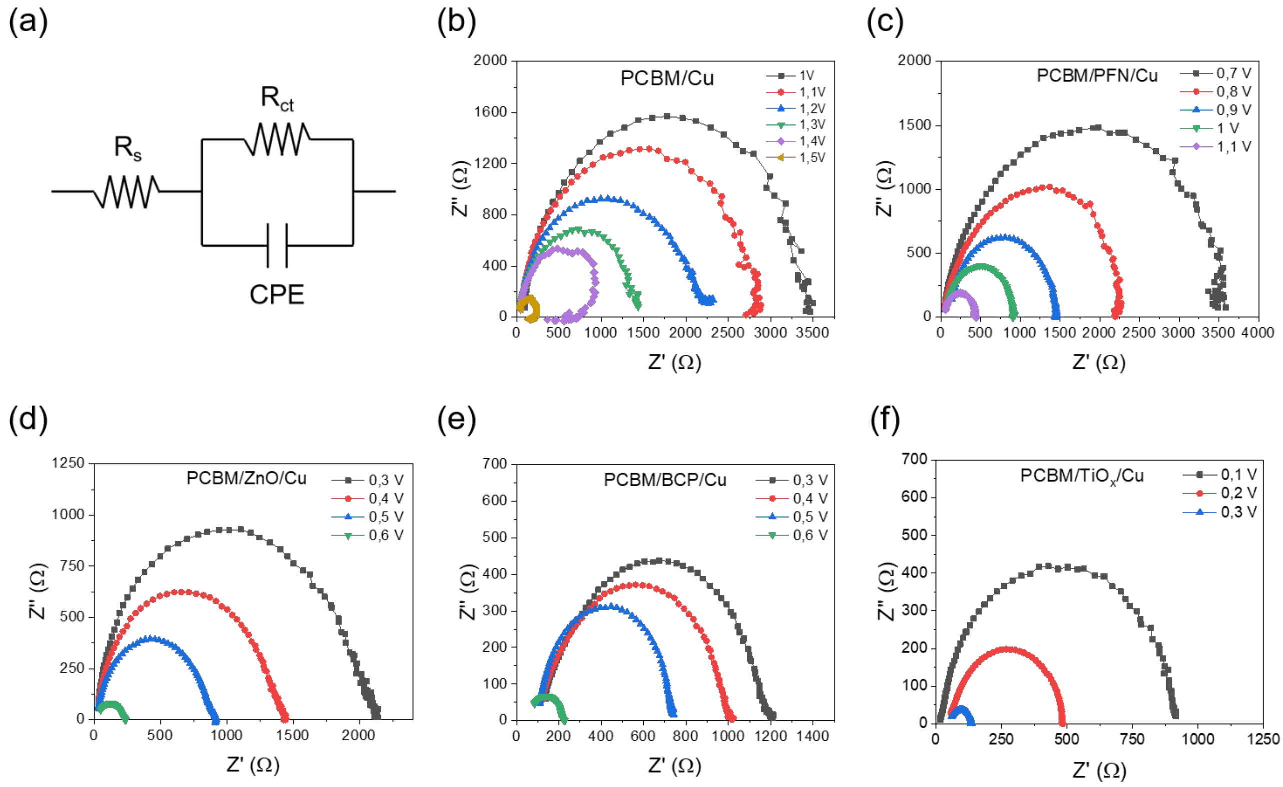

Figure S13. a) Equivalent circuit for fitting the impedance data. Impedance spectra for the degraded devices b) without CIL and with the CILs, including c) PFN, d) ZnO, e) BCP, and f) TiO_x, measured under dark conditions.

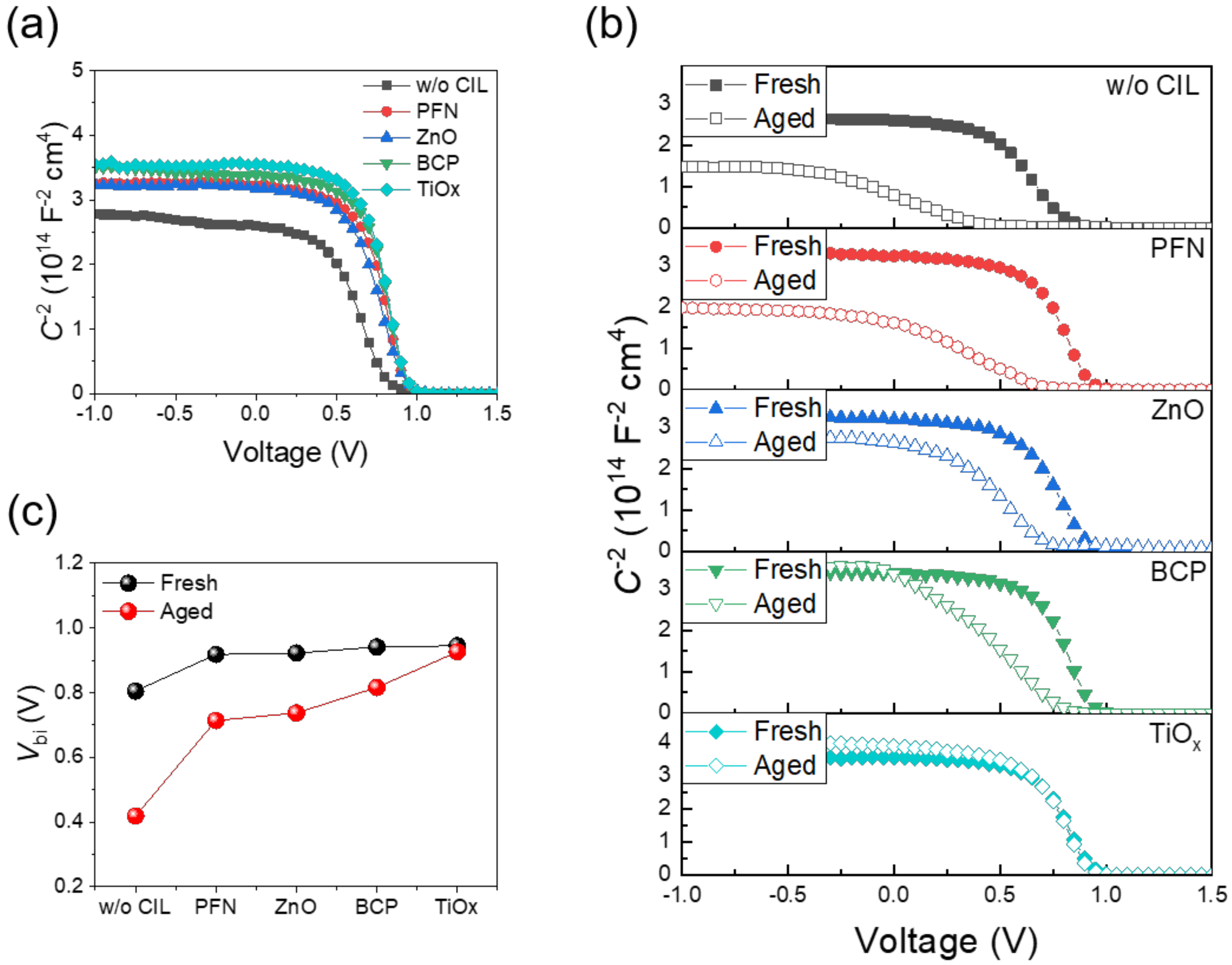

Figure S14. a) Mott-Schottky plots of the capacitance-voltage (C-V) characteristics for the fresh PSCs with CILs. b) Comparison data of fresh and degraded devices. c) V_{bi} values derived from the Mott-Schottky analysis.

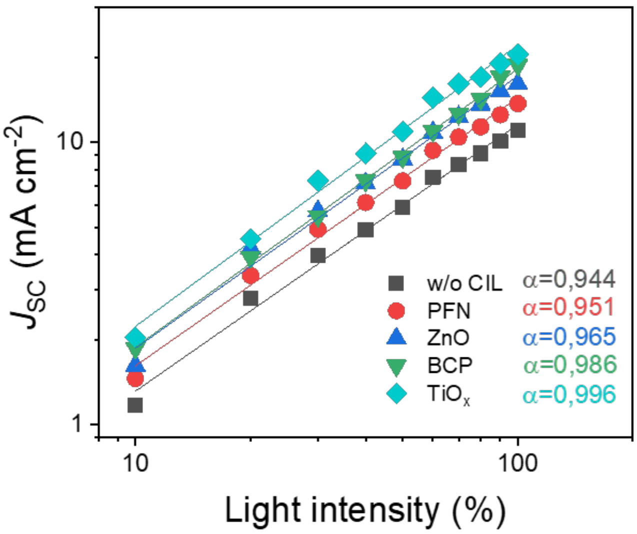

Figure S15. Dependence of J_{SC} on light intensity for degraded PSC devices with different CILs.

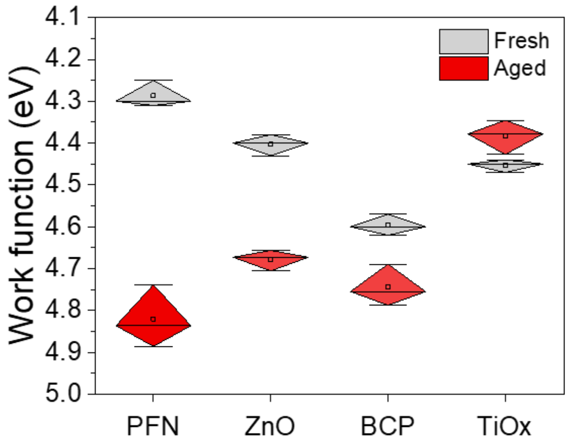

Figure S16. Dependence of J_{SC} on light intensity for degraded PSC devices with different CILs.

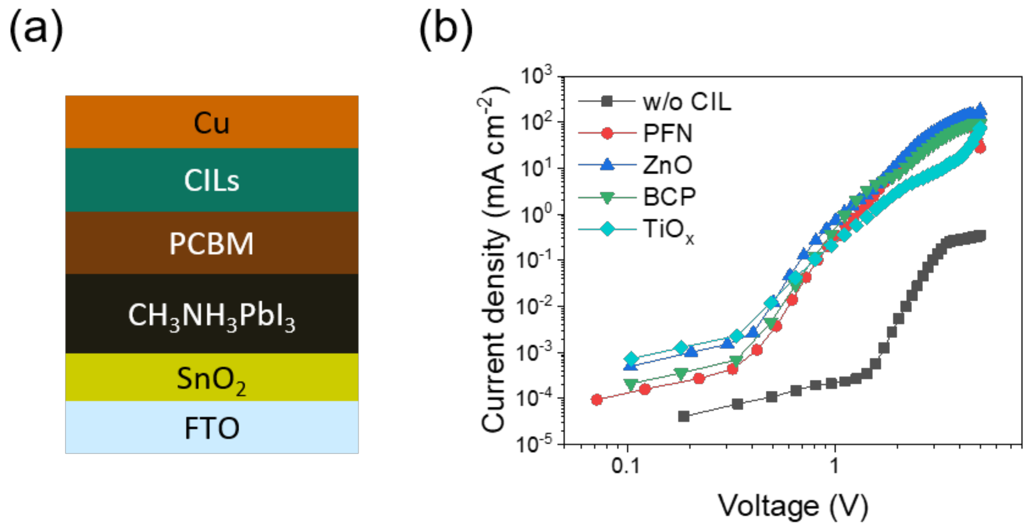

Figure S17. a) Device structure for electrical measurements of electron-only devices. b) Dark *J-V* characteristics of fresh electron-only devices with different CILs.

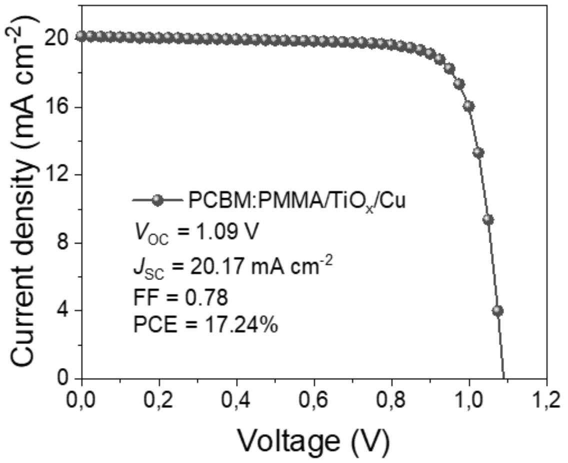

Figure S18. *J-V* characteristics of PSCs with PCBM:PMMA and thick TiO_x CIL.

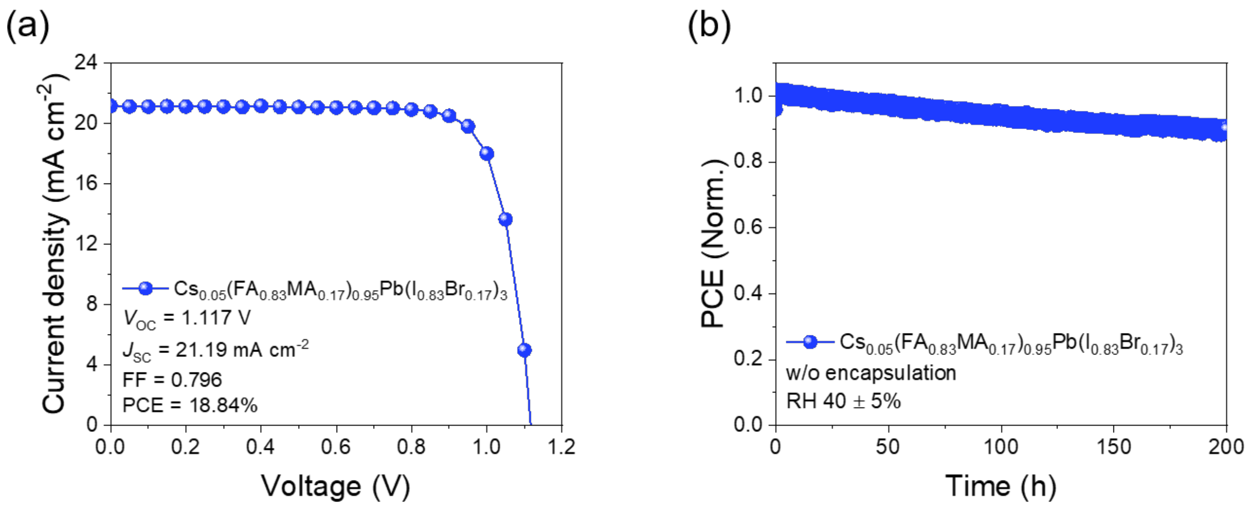

Figure S19. (a) *J*−*V* characteristics and (b) MPP tracking of triple cation PSCs with $Cs_{0.05}(FA_{0.83}MA_{0.17})_{0.95}Pb(I_{0.83}Br_{0.17})_3$ as a photoactive layer.

Table S1. Summary of the parameters calculated from the dark J-V characteristics of aged, electron-only devices ($FTO/SnO_2/MAPbI_3$/PCBM/CIL/Cu).

**CIL**	V_{TFL} **(V)**	N_d **(cm^{-3})**	E_{ch} **(meV)**
w/o CIL	2,247	5,07 X 10^{16}	312
PFN	1,754	3,96 X 10^{16}	252
ZnO	1,394	3,14 X 10^{16}	225
BCP	0,888	2,01 X 10^{16}	184
TiO_x	0,534	1,21 X 10^{16}	128

Table S2. Summary of the parameters calculated from the dark J-V characteristics of fresh, electron-only devices ($FTO/SnO_2/MAPbI_3$/PCBM/CIL/Cu).

**CIL**	V_{TFL} **(V)**	N_d **(cm^{-3})**	E_{ch} **(meV)**
w/o CIL	1,471	3,32 X 10^{16}	188
PFN	0,379	8,56 X 10^{15}	137
ZnO	0,404	9,13 X 10^{15}	155
BCP	0,369	8,33 X 10^{15}	141
TiO_x	0,335	7,57 X 10^{15}	77
